# LRP4 LDLα repeats of astrocyte enhance dendrite arborization of the neuron

**DOI:** 10.1186/s13041-020-00708-z

**Published:** 2020-12-10

**Authors:** Min Yan, Amin Guo, Peng Chen, Hongyang Jing, Dongyan Ren, Yanzi Zhong, Yongqiang Wu, Erkang Fei, Xinsheng Lai, Suqi Zou, Shunqi Wang

**Affiliations:** 1 School of Life Sciences, Nanchang University, Nanchang, Jiangxi China; 2 School of Basic Medical Sciences, Nanchang University, Nanchang, China; 3 Institute of Life Sciences, Nanchang University, Nanchang, Jiangxi China

**Keywords:** LRP4, Ldlα repeats, Dendrite arborization, Pyramidal neuron, Golgi staining, Primary culture

## Abstract

The low-density lipoprotein receptor-related protein 4 (LRP4) is essential for inducing the neuromuscular junction (NMJ) formation in muscle fibers, and LRP4 plays a critical role in dendritic development and synaptogenesis in the central nervous system (CNS). As a single transmembrane protein, LRP4 contains an enormously sizeable extracellular domain (ECD), containing multiple LDLα repeats in the N-terminal of ECD. LRP4 only with extracellular domain acts as a similar mechanism of full-length LRP4 in muscles to stimulate acetylcholine receptor clustering. In this study, we elucidated that LDLα repeats of LRP4 maintained the body weight and survival rate. Dendritic branches of the pyramidal neurons in *Lrp4-null* mice with LRP4 LDLα repeats residue were more than in *Lrp4-null* mice without residual LRP4 domain. Supplement with conditioned medium from LRP4 LDLα overexpression cells, the primary culture pyramidal neurons achieved strong dendritic arborization ability. Besides, astrocytes with LRP4 LDLα repeats residue could promote pyramidal neuronal dendrite arborization in the primary co-cultured system. These observations signify that LRP4 LDLα repeats play a prominent underlying role in dendrite arborization.

## Introduction

Synapses formed between neurons and target cells are the basis of brain function, and synaptic transmission is critical for learning, memory, and response to environmental changes. Furthermore, dysfunction of synapses involves various neuropsychiatric diseases, such as autism, schizophrenia, and epilepsy. In neuromuscular junction (NMJ) development and maintenance, LRP4 plays a vital role [1–3]. *Lrp4* null mice die at birth because of breath deficit, and biochemical studies have confirmed that LRP4 is a crucial regulator for NMJ formation [4]. It also is the receptor of Agrin and binds explicitly to neural Agrin, which promotes NMJ development [1, 3–6]. The single transmembrane protein LRP4 contains a small intracellular domain (ICD) and an enormous extracellular domain (ECD), and LRP4 ECD has eight LDLα domains, four β-propeller domains, and a domain for O-linked oligosaccharide modification. *LRP4*^*ECD/ECD*^ mice form partially functional NMJ, expressing only the ECD, without the transmembrane domain (TMD) and ICD [7]. The results from *LRP4*^*ECD/ECD*^ mice suggested that the TMD and ICD are not required for NMJ formation [5, 8, 9], and the LRP4 ECD domain plays a prominent role in the NMJ formation.

*Lrp4* is mainly expressed in the hippocampus, olfactory bulb, cerebellum, and neocortex, especially in the hippocampal postsynaptic membrane [10–12]. Early studies indicate that LRP4 is a protein present in the pyramidal neurons [12, 13]. Researchers found that LRP4 plays multiple roles in the CNS, involving hippocampal synaptic plasticity, excitatory synaptic transmission, fear regulation, and long-term enhancement [13, 14]. Furthermore, impaired synaptic plasticity and cognitive function deficits have been found in *Lrp4* knockout mice [13, 15]. Recently, Astrocytic LRP4 has been shown to regulate glutamatergic synaptic transmission by regulating adenosine triphosphate (ATP) release [11]. The role of the LRP4 extracellular segment in the brain is currently unclear.

In this study, two types of muscle-rescued *Lrp4-null* mice were used to identify the function of LRP4 LDLα: muscle-rescued *Lrp4*^*LacZ/LacZ*^ mice (*mr-Lrp4*^*LacZ*^) and muscle-rescued *Lrp4*^*mitt/mitt*^ mice (*mr-Lrp4*^*mitt*^). Astonishingly, we found some intriguing differences between *mr-Lrp4*^*LacZ*^ mice and *mr-Lrp4*^*mitt*^ mice, which were ignored or not paid special attention in previous research. LRP4 LDLα domain affects the survival rate and bodyweight of mice. Significantly, the LRP4 LDLα domain manipulates the dendritic arborization of pyramidal neurons in vivo and in vitro. These results indicate that the LRP4 LDLα domain plays a crucial role in developing CNS during dendritic branching.

## Materials and methods

### Animal

*Lrp4-null* mice (such as *Lrp4*^*mitt/mitt*^ and *Lrp4*^*LacZ/LacZ*^) died at birth because *Lrp4-null* mice could not move or breathe with NMJ deficits. *HSA-Lrp4* transgenic mice [9] cross with *Lrp4*^*mitt/*+^ mice (RRID: MMRRC_048465-UCD)[4] or *Lrp4*^*LacZ/*+^ mice [11] to generate *Lrp4-null* mice with muscle-rescued *Lrp4 *expression (*Lrp4*^*mitt/mitt*^*;HSA-Lrp4 Tg* and *Lrp4*^*LacZ/LacZ*^*;HSA-Lrp4 Tg,* briefly as *mr- Lrp4*^*mitt*^ and *mr-Lrp4*^*LacZ*^*,* respectively) can breathe and move freely [9, 13, 14, 16]. There is a stop code in the amino acid 377 in LRP4 in *Lrp4*^*mitt/mitt*^ mice [4]. *HSA-Lrp4* transgenic mice express exogenous wild-type *Lrp4,* specifically in skeletal muscle [9]. In *Lrp4*^*LacZ/*+^ mice, β-galactosidase protein cassette, including both stop code and a polyadenylation termination signal, was inserted into the downstream of *Lrp4* promoter [11].

Mice were housed in ventilated cages. Five or fewer adult mice were feed in each cage. Sufficient water and food were free intakes to mice, with a 12-h light/dark cycle, room temperature at 22 to 25 °C, and humidity is 50–60%. All experiments involving animals were conducted according to the “guidelines for the care and use of experimental animals” issued by Nanchang University. The Committee on the Ethics of Animal Experiments of the University of Nanchang approved the protocol (Permit Number: 2016–0002). For in vivo experiment, surgery was performed under sodium pentobarbital anesthesia (50 mg/kg, ip. injection), and all efforts were made to minimize suffering. After terminal experiments, mice were euthanized by carbon dioxide inhalation.

### Nissl’s staining

Brain slides were cut in 40 μm, washing with distilled water for 3 min. Dyeing in the staining buffer (0.2% Cresyl Violet solution) for 5 min at 60 °C. Then dehydrating slides with 50%, 75%, 90% ethanol (Cat.# A500737, Sangon Biotech) for 20 s. Putting the slides into 100% ethyl alcohol 3 times, each time for 20 s, transferring slides into xylene (Cat.# A530011, Sangon Biotech) 3 times, each time for 10 min. Samples were mounted in Hydromount (National Diagnostics, USA, HD-106).

### Golgi staining

Golgi staining was performed using the FD Rapid Golgi Stain™ Kit (FD NeuroTechnologies, USA, PK-401). Staining solution D, solution E, and ultra-pure water were mixed in a ratio of 1:1:2. Slides were incubated with staining solution at room temperature for 10 min and washed twice with ultra-pure water each time for 4 min. Transferring brain slides to the plate hole containing 50%, 75%, and 90% ethanol for 4 min each time, the brain slides were put into the holes containing 10 ml 90% ethanol or 100% ethanol for 3 times, 4 min for each time. Followed the samples were put into the xylene for 1 h. Images were randomly taken. Pyramidal neurons with clear dendritic branches were subjected to Sholl analysis by using ImageJ. The investigator who performed the analysis was blinded to genotypes.

### Cell culture and plasmid transfection

HEK293T (RRID: CVCL_0063) cells were cultured with media (DMEM + 10% Fetal bovine serum + 1% Pens/Strep) in a cell culture incubator at 37 °C and 5% CO_2_ and changed the medium once every 3 days. After the cell density reached about 60%, added the plasmids (pFLAG-CMV1 vector, pFLAG-CMV1-Lrp4-LDLα, or pFLAG-CMV1-Reelin) and polyethyleneimine (Cat.# 24314, Ploysciences) to 50 μl of serum-free DMEM culture medium according to the ratio of 3 μg of the plasmid to 15 μl of PEI, then mixed at room temperature. 20 min later, the mixture was added to HEK293T cells. After cultured in an incubator at 37 °C and 5% CO_2_ for 4–8 h, the culture medium was replaced with a new HEK293T culture medium. After 24 h, the conditioned medium collected from the supernatant was added into the primary neurons from the hippocampi of wild-type mice (DIV4). Primary neurons were changed half-medium every other day, and after 6 days (DIV10), the neurons were preformed to immunofluorescent staining.

### Immunoprecipitation (IP) and Western blotting

Anti-Flag beads (Cat. # M8823, Sigma Aldrich) were used to immunoprecipitate target protein from the cell culture medium. Western blotting was performed as described previously [17]. In brief, total proteins were extracted by RIPA. After SDS-PAGE, samples were transferred to the PVDF membrane (Millipore, USA) with transfer membrane apparatus (BIO-RAD, USA). The membrane was blocked by 5% milk in blocking buffer for 2 h. Wash the membrane with a washing buffer 3 times. Anti-Flag (Cat.# F1804, Sigma Aldrich) and anti-β-Actin (Cat.# ab8227, Abcam) primary antibody was added and incubated at 4 °C overnight. The HRP-labeled secondary antibody was added (1:2000, Abcam) to incubate at room temperature for 2 h and then washed 3 times. Luminata TM Crescendo Western HRP Substrate was added. Immunoreacted bands were captured by an enhanced chemiluminescence system (BIO-RAD, USA).

### Astrocyte and neuron co-culture

Primary cell separation and co-culture were performed as described previously [11] with minor modifications. The isolated hippocampi or the cerebral cortexes of E18 mice were cut into small pieces and digested in 0.25% trypsin at 37 °C for 10–15 min. For primary neuron culture, dissociated cells were resuspended in primary culture medium (Neurobasal + 1% GlutaMax™ + 5% FBS + 1% Pens/Strep) and plated onto poly-L-lysine–coated coverslips in 12 well-plates for 4 h. And then replacing medium with serum-free medium (Neurobasal + 1% GlutaMAX™ + 2% B27 + 1% Pens/Strep) and cytosine arabinoside (Cat.# HY-13605, Med ChemExpress) (10 μM) to inhibit glial proliferation. Half of the medium was changed every other day. For astrocyte culture, dissociated cells from the cerebral cortexes were resuspended in the plating medium (DMEM + 10% Fetal bovine serum + 1% GlutaMAX™ + 1% Pens/Strep) and plated into culture flasks for 3 d. The flasks were shaken at 250 rpm for 24 h to remove microglia and oligodendrocytes. Astrocytes were passaged every 3 d at a ratio of 1:3 and seeded onto coverslips before co-culture. After neurons were cultured 8 d (DIV8), three coverslips seeded astrocytes were placed with one neuron coverslip in one 35-mm dish containing serum-free medium for incubating 7 d (DIV15) until immunofluorescent staining. Co-culture primary neurons were also isolated from the cerebral cortexes, the same brain part as the astrocytes.

### Immunofluorescent staining

The coverslips of cultured neurons were fixed at room temperature for 20 min in 4% paraformaldehyde. After rinsing 10 min with phosphate-buffered saline (PBS) (0.01 M, pH 7.4) at room temperature, the coverslips were immersed in antibody blocking solution (10% donkey serum, 1% calf serum albumin, 0.5% Triton X-100 in PBS) at room temperature for 2 h. After this, the coverslips were rinsed with 0.01 M PBS at room temperature 3 times. The primary antibody (anti-β3-Tubulin antibody, Cat.# PA5-95875, Thermo Fisher Scientific) was diluted 1:1000 with an antibody blocking solution and was added into the coverslips for 4 °C overnight. The coverslips were washed with 0.01 M PBS at room temperature 3 times, each time for 10 min. The secondary antibody (anti-IgG, Cat.# 1832035, Invitrogen) was diluted 1:1000 with an antibody blocking solution, and then the coverslips were incubated at room temperature for 2 h in the dark. After washing 3 times for 10 min with PBST, samples were mounted in Hydromount (National Diagnostics). Images were collected with an Olympus fluorescence microscope (FSX100) and collapsed into a single image. Same areas as the Nissl staining, the brain slice was also used for anti-NeuN (Cat.# MAB377, Merck Millipore) staining, a similar procedure to the above.

### Experimental design and statistical analysis

The study was not pre-registered. For the assignment of experimental groups, no unique randomization methods were employed. Sample sizes and the number of cells were determined by experience and not by a statistical sample size calculation. The experiments reported in this work did not require institutional approval. Exclusion criteria were not pre-determined in this study. Data were statistically analyzed using GraphPad Prism 5.0 (RRID: SCR_002798, GraphPad Software, CA, USA), and the results were expressed as mean ± standard error (Mean ± SEM). A different person performed the analysis and experimental group assignments than the experimenter. Two-way ANOVA analyzed the measurement data; an independent sample T-test analyzed the comparison between groups. The mice numbers of independent experiments were noted in the figure legend. The difference was considered statistically significant, and P < 0.05 was considered statistically significant, P > 0.05 was considered no significant.

## Results

### LRP4 LDLα domain was necessary to keep body weight and survival rate

**Fig. 1:**
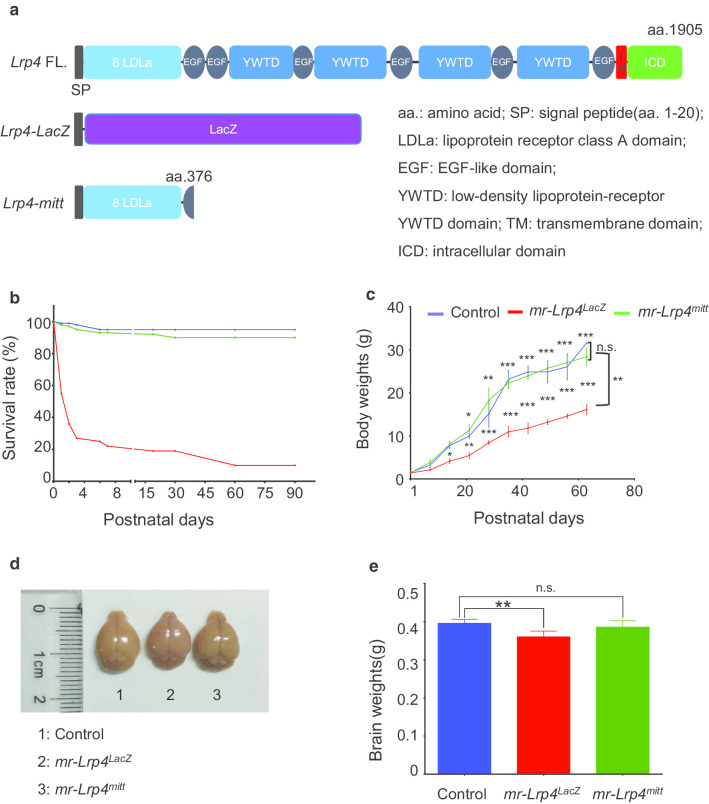
LRP4 LDLα domain is necessary to keep body weight and survival rate. **a** Schematic diagram of *Lrp4-LacZ* and *Lrp4-mitt*; **b** Comparing with *mr-Lrp4*^*mitt*^ and the control mice, the survival rate of *mr-Lrp4*^*LacZ*^ mice reduced. Control mice, n = 51; *mr-Lrp4*^*mitt*^ mice, n = 53; *mr-Lrp4*^*LacZ*^ mice, n = 39; **c** Compared with the other two types of mice, the bodyweight of *mr-Lrp4*^*LacZ*^ mice reduced. Multiple comparisons were made between the control and *mr-Lrp4*^*LacZ*^ mice (Above the curves) or between *mr-Lrp4*^*mitt*^ and *mr-Lrp4*^*LacZ*^ mice (Between the curves). All mice were male. Control mice, n = 21; *mr-Lrp4*^*LacZ*^ mice, n = 18; *mr-Lrp4*^*mitt*^ mice, n = 22; **d** Representative brain of the control, *mr-Lrp4*^*LacZ*^ and *mr-Lrp4*^*mitt*^ mice; **e** Brain weights of the three types of mice; Mice per type, n = 3. (Values are means ± SEM. Two-way ANOVA and t-test were used for analysis. n.s., not significant, * P < 0.05, ** P < 0.01, *** P < 0.001)

Bodyweight and survival rate of *mr-Lrp4*^*LacZ*^ mice, *mr-Lrp4*^*mitt*^ mice, and the control mice were monitored for more than 60 d. Compared with the control mice, the survival rate was significantly lower in the *mr-Lrp4*^*LacZ*^ group (Fig. 1b). The *mr-Lrp4*^*LacZ*^ mice gained significantly less body weight and brain weight (Fig. 1c-e). However, *mr-Lrp4*^*mitt*^ mice appeared healthy and were indistinguishable from the control mice. There was no significant difference between *mr-Lrp4*^*mitt*^ mice and the control mice in body weight, survival rate, and brain weight (Fig. 1b–e ). *mr-Lrp4*^*mitt*^ mice and *mr-Lrp4*^*LacZ*^ mice were all *Lrp4-null* mice, but there were significant differences in phenotype between *mr-Lrp4*^*LacZ*^ and *mr-Lrp4*^*mitt*^ mice. The *mr-Lrp4*^*mitt*^ mice remained the LDLα domain of LRP4 theoretically, as shown in Fig. 1a. Therefore, we speculate whether the LDLα domain played a particular function to keep mice alive and body weight, which leads to phenotypic differences between the two types of mice.

### The thickness of the cerebral cortex reduced in *mr-Lrp4*^*LacZ*^ mice

**Fig. 2:**
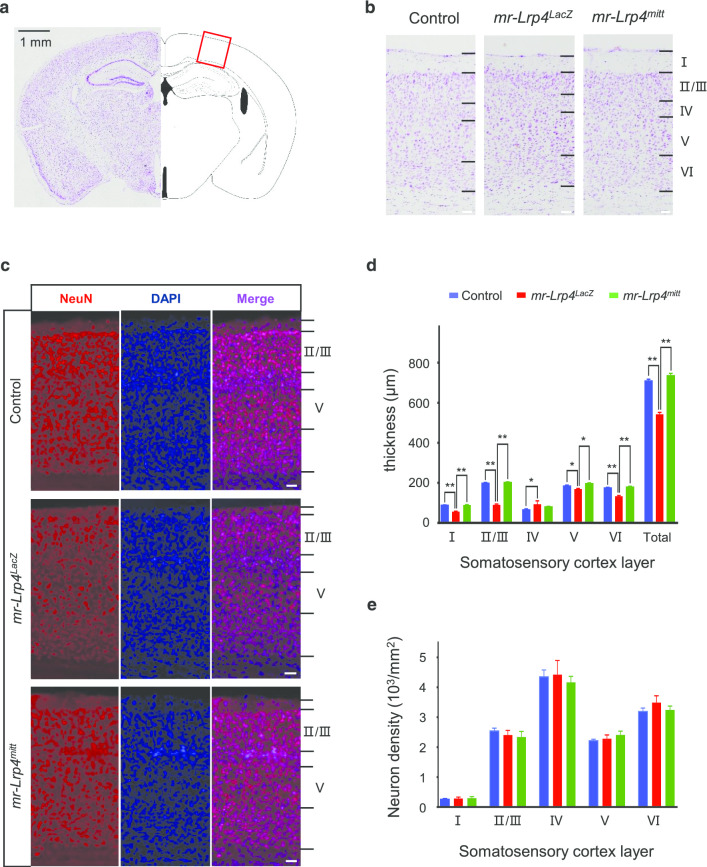
LRP4 LDLα domain affects the thickness of mouse cerebral cortex. **a** Schematic diagram of somatosensory cortex area selected in Nissl staining and neuron staining. **b** Representative images of Nissl’s staining of the control, *mr-Lrp4*^*LacZ*^*,* and *mr-Lrp4*^*mitt*^ mice brain; **c** Representative images of neuron staining (anti-NeuN) of the three types of mice brain; **d** The thickness of the somatosensory cortex in most layers (I, II/III, V and VI) decreased in *mr-Lrp4*^*LacZ*^ mice, except layer IV increased. Mice per group, n = 3; **e** The neuron density was not different in the somatosensory cortical indicated layers in the three types of mice. Mice per type, n = 3. (Scale bar = 50 μm; Values are means ± SEM. t-test was used for analysis. * P < 0.05, ** P < 0.01)

Nissl’s staining was carried out to observe the somatosensory cortex region (Fig. 2a) of the mice. The somatosensory cortex of *mr-Lrp4*^*LacZ*^ mice was markedly thinner in layer I, II/III, V, and VI than those of the control group, except layer IV increased. However, *mr-Lrp4*^*mitt*^ mice showed no difference from those of the control mice (Fig. 2b, d). The somatosensory cortical neuron density was similar in the three types of mice (Fig. 2c, e). In light of the remarkable difference in the thickness of *mr-Lrp4*^*LacZ*^ mice compared with *mr-Lrp4*^*mitt*^ or the control mice, we speculated that the LDLα domain of LRP4 maintained the typical structure of the cerebral cortex.

### The dendritic branches of pyramidal neurons increased in *mr-Lrp4*^*mitt*^ mice

**Fig. 3:**
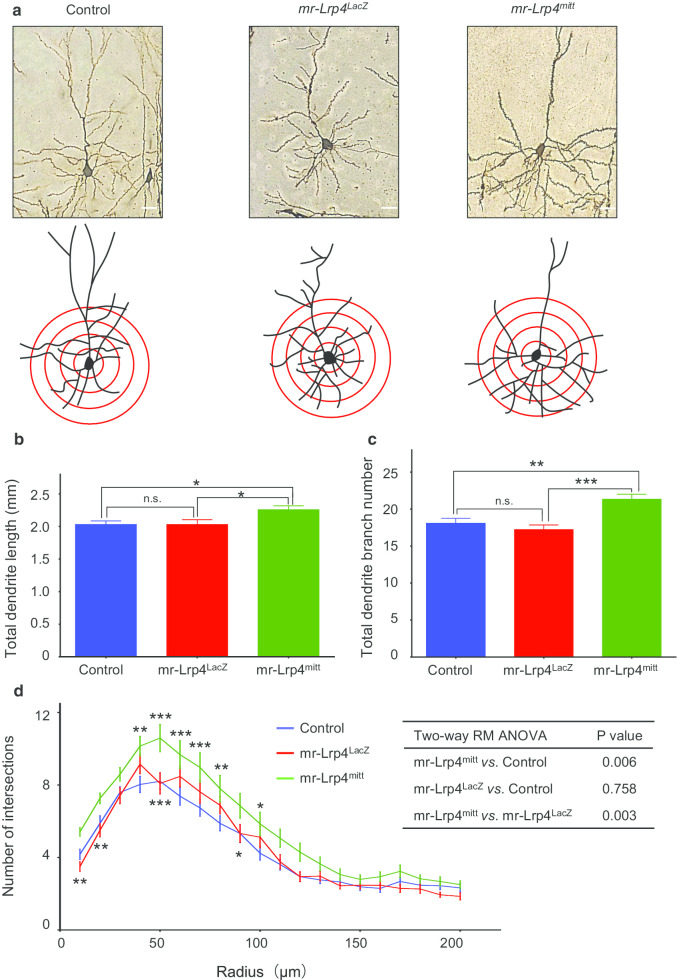
LRP4 LDLα domain enhances the dendrite arborization of neurons in *mr-Lrp4*^*mitt*^ mice. **a** Representative images of Golgi staining the neurons in the cerebral cortex of the control, *mr-Lrp4*^*LacZ*^*,* and *mr-Lrp4*^*mitt*^ mice; using the ImageJ plugin for Sholl analysis; **b** The total dendrite length of neurons in *mr-Lrp4*^*mitt*^ mice increased; **c** The total dendrite branch number of neurons in *mr-Lrp4*^*mitt*^ mice increased. **d** Sholl analysis of dendrite branch number of the three types of mice. Multiple comparisons were made between *mr-Lrp4*^*mitt*^ and the control mice (Above the curves) or between *mr-Lrp4*^*mitt*^ and *mr-Lrp4*^*LacZ*^ mice (Below the curves). Control mice, n = 6; *mr-Lrp4*^*LacZ*^ mice, n = 3; *mr-Lrp4*^*mitt*^ mice, n = 3; Neuron per group, n = 34. (Scale bar = 30 μm; Values are means ± SEM. t-test and two-way ANOVA were used for analysis. n.s., not significant, * P < 0.05, ** P < 0.01, *** P < 0.001)

Golgi staining was performed to identify the morphological structure of cerebral cortex pyramidal neurons in *mr-Lrp4*^*LacZ*^, *mr-Lrp4*^*mitt*^ mice, and the control mice. The complexity of pyramidal neurons in layer 4–5 of the somatosensory cortex was analyzed by Sholl analysis. Sholl analysis centered on the pyramidal neuronal cell body, a series of concentric circles were drawn and obtained the number of intersections of pyramidal neuronal processes varying with the cell body’s distance. Compared with the control mice, there was no difference in total dendrites length presented in *mr-Lrp4*^*LacZ*^ mice, but the total dendrite length in *mr-Lrp4*^*mitt*^ mice was longer (Fig. 3b). In *mr-Lrp4*^*LacZ*^ mice, we observed no significant difference in the somatosensory cortex pyramidal neurons’ total dendrites branches than the control group (Fig. 3c, d). More dendrite branches were showed in *mr-Lrp4*^*mitt*^ mice than the control mice (Fig. 3c, d). These indicated that the LRP4 LDLα domain might play a prominent role in promoting dendritic arborization.

### LRP4 LDLα domain increased dendrite arborization in vitro

**Fig. 4:**
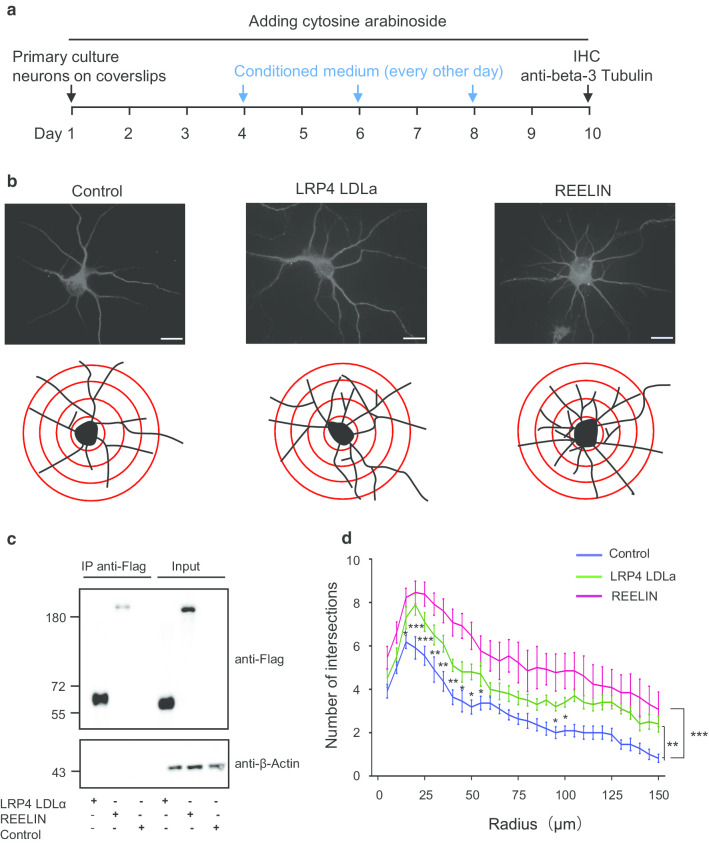
LRP4 LDLα domain increases the dendrites arborization in primary culture neurons. **a** Schematic diagram of time course dealing with the primary culture neurons. pFLAG-CMV1-Lrp4-LDLα, pFLAG-CMV1-Reelin, and pFLAG-CMV1 vector plasmids were transfected into HEK293T cells, and then the conditioned medium was collected to add into primary cultured wild-type mice neurons, respectively. In panel B-D, pFLAG-CMV1 vector group, pFLAG-CMV1-Lrp4-LDLα group, and pFLAG-CMV1-Reelin group were abbreviated as control, LRP4 LDLα and REELIN, respectively; **b** Representative images of neurons staining in primary culture; Using the ImageJ plugin tracing the neurite branches; **c** Western blotting check the expression of the plasmids after transfected into HEK293T cells. Anti-Flag for LRP4 and REELIN immunoprecipitation from the cell culture medium. The input was from the cell lysis; **d** LRP4 LDLα domain improved the number of dendritic branches in primary neurons. The ImageJ plugin was used for Sholl analysis. Multiple comparisons with two-way ANOVA analyses were made between the LRP4 LDLα group and the control (between the curves). (Scale bar = 30 μm; Wild-type mice, n = 8; Neuron number, control n = 11, LRP4 LDLα n = 10 and REELIN n = 13. Values are means ± SEM. * P < 0.05, ***P < 0.001, *** P < 0.001)

To assess whether the LRP4 LDLα domain played a role in the pyramidal neuronal dendritic arborization, we transfected pFLAG-CMV1-Lrp4 LDLα into HEK293T cells, pFLAG-CMV1-vector as the negative control, and pFLAG-CMV1-Reelin plasmid was a positive control. REELIN and LRP4 LDLα secreted into the cell culture medium were confirmed by immunoprecipitation (IP) assay (Fig. 4c). The conditioned medium collected from HEK293T cell culture was added to the primary cultured neurons isolated from wild-type mice’s hippocampi. The number of dendrites branches increased when the conditioned medium containing LRP4 LDLα, compared with the negative control (Fig. 4b, d). In *mr-Lrp4*^*mitt*^ mice, the LDLα domain was secreted because of LRP4 lack of transmembrane domain, similar to the plasmid expression in HEK293T cells.

**Fig. 5:**
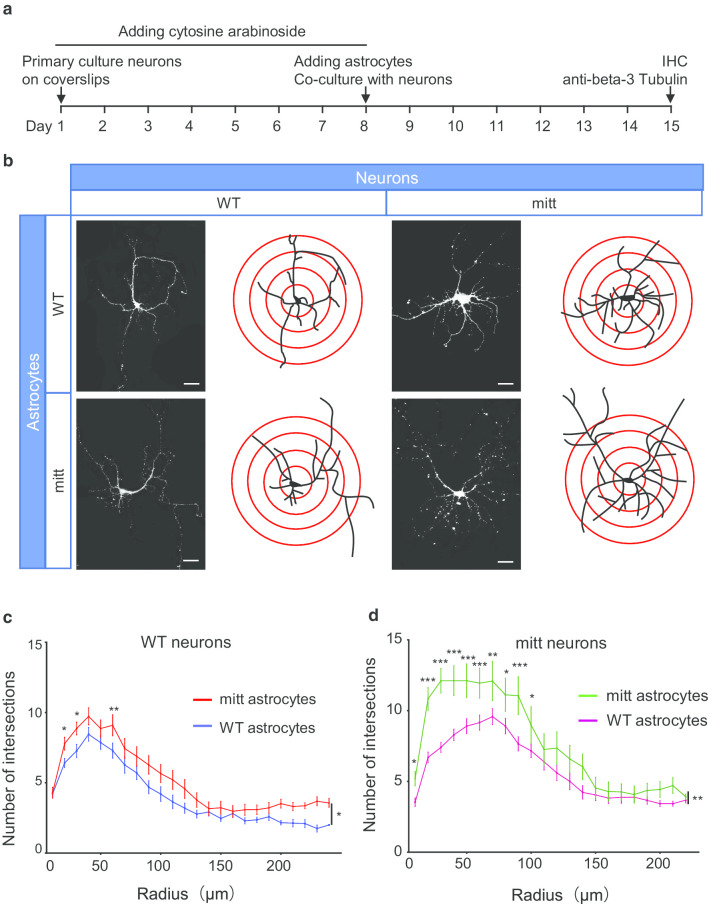
LRP4 LDLα domain in astrocytes promotes dendrite arborization in primary co-cultured neurons.** a** Schematic diagram of time course dealing with co-culture neurons with astrocytes. **b** Representative images of neurons staining in co-culture. Primary neurons of *mr-Lrp4*^*mitt*^ mice or wild-type mice were co-cultured with astrocytes from *mr-Lrp4*^*mitt*^ mice or wild-type mice; Using the ImageJ plugin tracing the neurite branches; **c** Co-cultured with astrocytes from *mr-Lrp4*^*mitt*^ mice, neuronal dendrite branch number of wild-type mice was more than co-cultured with astrocytes from wild-type mice. A similar difference is present in the co-cultured neurons of *mr-Lrp4*^*mitt*^ mice with astrocytes from *mr-Lrp4*^*mitt*^ mice or wild-type mice. Using the ImageJ plugin for Sholl analysis. (Scale bar = 30 μm; Wild-type mice, n = 7; *mr-Lrp4*^*mitt*^ mice, n = 8; Neuron per group, n = 52. Values are means ± SEM. Multiple comparisons with two-way ANOVA analyses were also made between the groups. * P < 0.05, ** P < 0.01, *** P < 0.001)

Studies showed that *Lrp4* was expressed in neurons and astrocytes, and *Lrp4* knockout in astrocytes suppressed glutamatergic release by increasing ATP release [11]. To explore whether the LRP4 LDLα promoting dendritic arborization were from neuron or astrocyte, we co-cultured the astrocytes with the neurons from wild type mice or *mr-Lrp4*^*mitt*^ mice. Pyramidal neurons of wild type mice showed more branches being co-cultured with astrocytes from *mr-Lrp4*^*mitt*^ mice than astrocytes from wild type mice (Fig. 5b, c). Without controversy, the *mr-Lrp4*^*mitt*^ mice pyramidal neurons boosted more dendritic arborization co-cultured with astrocytes from *mr-Lrp4*^*mitt*^ mice than astrocytes from wild type mice (Fig. 5b, d). The data further confirmed that free LRP4 LDLα promoted dendrite arborization of pyramidal neurons.

## Discussion

Here we demonstrated that the survival rate and bodyweight of *mr-Lrp4*^*LacZ*^ mice were lower than *mr-Lrp4*^*mitt*^ mice and the control mice. The brain tissue also was smaller. Second, the somatosensory cortex was thinner in layer I, II/III, V and VI of *mr-Lrp4*^*LacZ*^ mice than those of the control group, except layer IV increased. In *mr-Lrp4*^*mitt*^ mice, LRP4 has the LDLα domain, but this domain was missing in *mr-Lrp4*^*LacZ*^ mice. We speculated that these changes might be related to the function of the LDLα domain. Therefore, to clarify the LRP4 LDLα domain’s role, we performed Golgi staining to observe pyramidal neuronal dendritic branches. The results showed more dendritic branches in the pyramidal neurons of *mr-Lrp4*^*mitt*^ mice than in the control group. On the contrary, there was no difference between *mr-Lrp4*^*LacZ*^ mice and the control group.

Third, neuronal cells supplementary with the conditioned medium collected from HEK293T cells, which were transfected with LRP4 LDLα plasmids, had more dendritic branches than the control group. When neurons were co-cultured with astrocytes of *mr-Lrp4*^*mitt*^ mice, the number of dendritic branches increased. These results indicated that the LDLα domain of LRP4 promoted more dendritic arborization in pyramidal neurons.

N-terminal region of LRP4 is an enormous ECD, which has eight LDLα domains (class A repeats), four β-propeller domains (class B repeats). LRP4 has a fundamental role during the formation, maintenance, and regeneration of the NMJ [18]. LRP4 β1 domain binds with Agrin to form the Agrin-LRP4 binary complex to activate acetylcholine receptor (AChR) clustering in NMJ [5, 6]. Study results showed that treatment ecto-LRP4 (Lrp4 ECD only) into myotubes, ecto-LRP4 increases the number of Agrin-induced AChR clusters. It indicates that soluble ecto-LRP4 is sufficient to serve as a receptor for Agrin to initiate AChR clustering pathways. Ecto-LRP4 acts via a similar mechanism of full-length LRP4 in muscles to stimulate AChR clustering [1]. In this experiment, the release of LDLα domain in *mr-Lrp4*^*mitt*^ mice may promote the increase of pyramidal neuronal branches.

LRP4 plays a crucial role in CNS, including maintaining synapses, especially in synaptic transmission [19, 20]. Studies demonstrated that in the brain, glutamate release is reduced in lacking *Lrp4* mice. *Lrp4 *knockout astrocytes suppress presynaptic glutamatergic release by increasing ATP release. ATP released from astrocytes is converted to adenosine that activates adenosine A1 receptors in glutamatergic pre-synapses. Herein, synaptic plasticity is affected [11].

Besides, LRP4 plays a role in dendritic development and synaptogenesis in the CNS. *Lrp4* knockdown or deficiency in embryonic cortical and hippocampal neurons causes a reduction in density of primary dendrites [20, 21], and Agrin induces an LRP4-independent increase in dendritic branch complexity [21, 22]. *Lrp4* knockdown by in utero electroporation of *Lrp4* miRNA also results in neurons with fewer primary dendrites in the developing cortex and hippocampus in vivo [20]. Furthermore, overexpression of *Lrp4* in these cultured neurons has the opposite effect inducing more but shorter primary dendrites [20]. Karakatsani et al*.* study [20] showed that the dendritic branching of the primary culture neurons significantly reduces upon *Lrp4* knockdown (DIV3 to DIV12), and the differences disappear during the maturation period (DIV12 to DIV21). In this study, *mr-Lrp4*^*LacZ*^ mice did not exhibit a difference in dendritic arborization than the control mice. There may be a mechanism to compensate for *Lrp4* knockdown or deletion in vitro and in vivo.

Lacking LRP4 ICD in *mr-Lrp4*^*mitt*^ mice is indeed the difference between *mr-Lrp4*^*mitt*^ and the control mice. This result suggests two main possible mechanisms. The LRP4 ICD may inhibit dendritic arborization, or LRP4 LDLα may promote the dendrite arborization. LRP4 LDLα in *mr-Lrp4*^*mitt*^ mice is the big difference between *mr-Lrp4*^*mitt*^ and *mr-Lrp4*^*LacZ*^ mice. Therefore, we consider that soluble LDLα promotes the dendrite arborization in *mr-Lrp4*^*mitt*^ mice. Pohlkamp et al*.* [14] indicated that the soluble LRP4 extracellular segment may negatively impact the other receptors in *Lrp4*^*ECD/ECD*^ mice. However, *Lrp4*^*ΔICD/ΔICD*^ mice that maintain the transmembrane have no such effect. The possibility of LRP4 LDLα secreted by cells was verified by IP assay from the cell culture medium (Fig. 4c).

Pyramidal neuronal LRP4 LDLα domain may be functional via a cell-autonomous manner in *mr-Lrp4*^*mitt*^ mice. *Lrp4* is mainly expressed in astrocytes [11], and recent research found that *Lrp4* also is expressed in neuron stem/progenitor cells [23] and neurons at a low level [20]. It is hard to distinguish the distinct effects of pyramidal neuronal LDLα domain and astrocyte-derived LDLα domain on pyramidal neuronal dendrite arborization in this study. It is an enhancement of astrocytic LRP4 LDLα on pyramidal neuronal dendritic arborization. If *Lrp4*^*flox/flox*^ mice build up *Lrp4* knockout in specific cells, primary culture neurons from astrocyte knockout *Lrp4* mice may eliminate astrocytic effect *Lrp4* in co-culture assay.

Overall, our data point to functional links between the LRP4 LDLα domain in modulating dendritic branching in developing CNS neurons. LRP4 LDLα domain promoted more dendritic branch formation. LRP4 LDLα domain binds with DKK1, Sclerostin, ApoE, Gremlin1, Wise. DKK1, Sclerostin, and Wise are factors that inhibit Wnt signaling by binding LRP5/6 [24–26]. DKK1 mutation causes the phenotypes of a double ridge and polysyndactyly [27]. Mutation of Sclerostin results in sclerosteosis [28]. Moreover, LRP4 ECD enhances sclerostin-mediated inhibition of Wnt/β-catenin signaling [29]. *Lrp4* mutation increases serum sclerostin levels in osteocytes [16]. However, the LRP4 interaction’s significance with the Wnt signaling pathway in the brain remains unclear [30]. LRP4 and Wise interaction is revealed to regulate the patterning and formation of gland development [31]. ApoE can bind with LRP4 [8, 23, 32, 33]. Moreover, ApoE, as one of the LRP4 ligands, is essential for developing the nervous system, regulating synaptic plasticity, neuroprotection, and the innervation of the muscle [8]. LRP4 interaction with ApoE promotes Aβ uptake [34]. ApoE also interacts with Reelin [8] and inhibits Reelin boost dendritic arborization [35–38]. Therefore, we speculated that free LRP4 LDLα in *mr-Lrp4*^*mitt*^ mice might promote dendritic arborization by relieving the inhibition of ApoE on the Reelin.

## Data Availability

The datasets used or analyzed during the current study are available from the corresponding author on reasonable request.

## Abbreviations

AChR: Acetylcholine receptor
ATP: Adenosine triphosphate
CNS: Central nervous system
DIV: Days in vitro
DMEM: Dulbecco’s modified eagle medium
ECD: Extracellular domain
ICD: Intracellular domain
IP: Immunoprecipitation
LRP4: Low-density lipoprotein receptor-related protein 4
NMJ: Neuromuscular junction
PBS: Phosphate-buffered saline
TMD: Transmembrane domain
